# Measuring healthy behaviours using the stages of change model: an investigation into the physical activity and nutrition behaviours of Australian miners

**DOI:** 10.1186/s13030-017-0115-7

**Published:** 2017-12-05

**Authors:** Sarah J. Lacey, Tamara D. Street

**Affiliations:** Wesley Medical Research, PO Box 499, Toowong, QLD 4066 Australia

**Keywords:** Behaviour change, Intervention design, Nutrition, Obesity, Physical activity, Stages of change

## Abstract

**Background:**

Obesity is one of the fastest growing modern day epidemics affecting preventable disease and premature deaths. Healthy lifestyle behaviours, such as physical activity and nutritional consumption, have been shown to reduce the likelihood of obesity and obesity related health risks. Originally designed for measurement of unhealthy behaviours, the Stages of Change model, describes ‘precontemplators’ as individuals who engage in the unhealthy behaviour, are unaware that their behaviour is problematic, and are resistant to change. The aim of this study was to refine and assess the measures of the Stages of Change model in order to achieve a concise and reliable classification of precontemplators, in the context of healthy behaviours.

**Methods:**

Eight hundred and ninety-seven employees participated in a health survey measuring current health behaviours and stage of change. This study compared a traditional precontemplation measure to a modified version in the assessment of two healthy behaviours: physical activity and fruit and vegetable consumption.

**Results:**

The modified measure was more accurate and captured fewer individuals currently meeting the guideline for both physical activity and nutrition, compared to the traditional measure of stages of change. However, across all stages of change, the measure incorrectly classified some employees with regards to meeting health guidelines.

**Conclusions:**

When applied to healthy behaviours, the stages of change measure for precontemplation should be further refined to reflect knowledge that the behaviour is unhealthy, and apathy to change. Additionally, measures should define health guidelines to increase reliable classification across all stages of change. The findings can be applied to inform the design and implementation of health promotion strategies targeting obesity related lifestyle behaviours in the general population.

## Background

Obesity, as defined by abnormal or excessive fat accumulation, has been associated with increased risk of chronic diseases; including cardiovascular disease, type 2 diabetes, musculoskeletal disorders, and some cancers [1]. The World Health Organization (WHO) refers to obesity as an ‘epidemic disorder’ [1]. Encouragingly, research has shown that the risk of obesity and related health disorders can be significantly reduced through healthy lifestyle behaviours, such as being physically active and maintaining a healthy diet [2]. Often, the scientific community has focused on treatment of obesity related disorders, most often in clinical samples [3]. More preventative work is needed in the area of behaviour change research applying obesity *prevention* strategies targeting lifestyle behaviours pertinent to the *general population*.

Obesity *prevention* strategies usually focus on communicating the benefits of a healthy lifestyle (e.g. The ‘Swap It, Don’t Stop It’ campaign’; [4]). Despite the benefits of a healthy lifestyle being largely well-known in western countries, the prevalence of obesity continues to rise. This trend suggests that the current mass education approach may, by itself, be ineffective in prompting behaviour change in the general population. Adults who continue to engage in unhealthy lifestyle behaviours considerably increase their risk of obesity, obesity related health risks, and premature death [5]. It is therefore imperative for health professionals to understand behaviour change models that explain variation in lifestyle behaviours in order to design effective obesity prevention behaviour change interventions.

### Behaviour change

Achieving and maintaining lifestyle behaviour changes are a multifaceted process encompassing an individual’s motivation for, and preparedness to, change. Health behaviour change typically requires a significant life event, such as the illness or death of a loved one and a newfound understanding of the consequences of the problematic behaviour, to prompt the change [6]. Hence, achieving lifestyle behaviour change is particularly difficult for ‘non-clinical’ individuals that have not been diagnosed with a specific illness or experienced a motivating life event. Arguably, it is this group of ‘precontemplators’ who lack the understanding of the consequences of their behaviours, self-efficacy, or motivation to change that would be the least likely to volunteer to participate in an obesity prevention intervention. To facilitate health practitioners in assisting precontemplators, a reliable measure to identify individuals who are not meeting health guidelines and not contemplating improving their health behaviours is needed.

### The importance of theory

Scientific research has shown that health behaviour change programs are most effective when they are guided by a theoretical construct [7–10]. Surprisingly, behavioural change interventions have seldom referred to theory in the design phase of practical interventions, rather than simply applying theoretical constructs as an outcome measure [11]. Hence, the ability to dissect and evaluate such behavioural change interventions is limited, and replication of successful results becomes less likely [12].

One of the most commonly applied models of behaviour change is Prochaska and DiClemente’s Stages of Change model. This model has been well validated in the literature [9, 13]. A major benefit of the Stages of Change model is its potential concision of assessment. This makes it attractive to health practitioners compared with other models of behaviour change, such as the Health Belief Model [14] and Theory of Planned Behaviours [15], that typically require assessment of a multitude of variables for the purpose of categorisation.

### The stages of change model

Studies of behaviour change have shown that modifying behaviour typically requires an individual to move through a series of stages of preparedness to change [16]. Prochaska and colleagues’ Stages of Change model [5, 17, 18] (which forms part of the broader Transtheoretical Model of Behaviour Change) assesses an individual’s readiness to change a health behaviour. Although there has been debate regarding the validity of the Stages of Change Model as a stand-alone theory outside of the context of the Transtheoretical Model (e.g. [19]), the majority of studies to date apply the model in this way [20]. The model is comprised of five stages that represent incremental increases in preparedness to change: (i) precontemplation – the individual is unaware of the consequences of their behaviour and resistant to change; (ii) contemplation – the individual is aware of the consequences of their behaviour and open to change; (iii) preparation – the individual shows anticipation and willingness to change within the next six months; (iv) action – the individual is in the process of changing their behaviour and shows enthusiasm and momentum; and (v) maintenance – the individual has sustained the new behaviour for more than six months and shows perseverance in maintaining the change.. In some versions of the model a sixth stage, ‘termination’ – which refers to the cessation of an unhealthy behaviour with no temptation to relapse – is included following the ‘maintenance’ stage.

Fundamentally, the model assesses change as a temporal dimension occurring over time rather than a singular event. The typical example of such a temporal change is an individual’s transition between smoking and non-smoking behaviours. That is, in assessing behaviour it is important to consider the process of preparing to change over time as well as the behaviour itself [21]. Although the time it takes an individual to progress from one stage to the next is variable, the processes required to advance, including decisional balance and self-efficacy, are not. It is estimated that only a minority (usually less than 20%) of a population is prepared to take action to change a specific health behaviour at any given time [16].

The strength of the Stages of Change model lies in its capacity to match the stage of each individual with specific intervention strategies [22]. Recently, the Stages of Change model has been successfully applied in the development of effective semi-tailored health interventions including smoking cessation [23], condom use [24], and fruit and vegetable consumption [25]. Moreover, interventions based on the Stages of Change model have been associated with greater participation when compared with other theory based interventions. This may be a result of the semi-tailored nature of the approach being perceived as a personalised program and appealing to the wider population [12].

### Measures of stages of change variables

Applying the Stages of Change model in an obesity prevention intervention requires a reliable and valid measure [13]. The most prominent measure within the literature is the University of Rhode Island Change Assessment Scale (URICA) [26]. The URICA includes 32-items measured on a 5-point likert scale. While some studies have condensed the stages of change assessment to a single item measure [20], little reporting has been done on the validity of such measures. Nevertheless, concision in assessment is preferred in applied settings where time constraints commonly exist (e.g. workplace settings [27]).

Since the Stages of Change model was developed for understanding unhealthy behaviours, this influences the wording of its associated measures. As such, adjustments may be required in order to apply both the model and associated measures for the assessment of healthy behaviours. For example, the first URICA item for precontemplation states, *“As far as I’m concerned, I don’t have any problems that need changing”.* When applied to the context of unhealthy behaviours it is clear that a positive response to the item would indicate a lack of awareness of the problem behaviour. However, when applied to a healthy behaviour such as physical activity, an individual who has sustained the healthy behaviour over a six month period, (representing the ‘maintenance’ phase of the model), may respond positively to this item. Therefore, in order to successfully apply the Stages of Change model to healthy behaviours for the purpose of obesity risk management interventions, it is imperative that the model be adjusted to accurately assess the stage constructs in the context of healthy behaviour changes.

### Project aim

According to the Stages of Change model, ‘precontemplators’ both engage in the unhealthy behaviour, and are unaware that their current behaviour is problematic. Therefore, a measure of those within the ‘precontemplation’ phase of the Stages of Change model should capture individuals that do not engage in the healthy behaviour (such as adequate physical activity and fruit and vegetable consumption). The aim of this study was to compare two concise survey measures of Prochaska and DiClemente’s [6] ‘precontemplation’ phase of stages of change in the assessment of healthy behaviours in a general population (i.e. non-clinical) workplace sample. The authors hypothesised that the modified measure of precontemplation, which expressed knowledge of a need to change the health behaviour but apathy to do so, would more accurately capture precontemplators’, who did not meet the WHO guideline for physical activity or nutrition [2], compared with the traditional measure of precontemplation.

## Method

### Participants

A cluster sample of 897 employees was recruited from selected work units within a large mining company operating in rural Australia. Participants were predominantly male (73.60%, *n* = 658), married or partnered (64.7%, *n* = 559), and predominantly aged between 25 and 44 years (58.9%, *n* = 474).

### Materials

#### Health survey

A self-report health survey was administered onsite to measure physical activity and nutrition health behaviours, and theoretical stage of change. The health related survey items replicated previously validated items in the Australian Bureau of Statistics Australian Health Survey [28]. Physical activity was measured as the total amount of minutes an individual engaged in moderate physical activity over a period of seven days. Nutrition was measured as the average quantity of daily serves of vegetables and fruit. Consistent with the WHO guideline [2], participants were classified as meeting the physical activity guideline if they reported engaging in at least 150 min of moderate exercise over a period of seven days. Participants were classified as meeting the nutrition guideline if they reported consuming an average minimum quantity of five serves of vegetables and two serves of fruit daily. The measure of theoretical stage of change based on the model by Prochaska and DiClemente [6] was modified to incorporate an additional ‘precontemplation’ stage response that read, *“I know I should improve my [exercise / eating] habits but I don’t intend to”*. Response options for all stages are presented in Table 1.

**Table 1:** Health Survey Stages of Change Survey Measures

Stages of Change	Survey Measure	
Maintenance	I took action more than 6 months ago to change my [exercise / eating] habits and I’m working hard to maintain this change	
Action	I am doing something to improve my [exercise / eating] habits	
Preparation	I have definite plans to improve my [exercise / eating] habits in the next month	
Contemplation	I’m seriously intending to improve my [exercise / eating] habits in the next 6 months	
Precontemplation	Traditional Measure As far as I’m concerned my [exercise / eating] habits don’t need changing	Modified Measure I know I should improve my [exercise / eating] habits but I don’t intend to

### Procedure

#### Health survey

During visits by the research team to the mining company, employees operating in selected representative work units were invited by their supervisor to participate in the health survey. Supervisors were not made aware of whether employees accepted the invitation to voluntarily participate.

#### Data analysis

All analyses were conducted using IBM SPSS version 21. Analysis of results involved chi square testing with post-hoc *Cramer’s V* analyses and cross tabulation analyses to compare the proportion of participants who met the guidelines for physical activity and nutrition in each stage of change.

## Results

The raw data was reviewed for accuracy of data entry, quantity and patterns of missing values. Of the 897 survey responses received, two participants were excluded from analysis; one participant was found to have completed only the demographic items of the survey whilst another participant failed to complete the theoretical stages of change questions. From the remaining 895 participants, a Little’s MCAR analysis confirmed that missing data occurred at random, χ^2^(20) = 17.43, *p* = .625. Survey responses that included missing data were excluded based on a pairwise deletion. No further data imputation occurred.

The demographic characteristics of survey participants has been summarised in Table 2. The worksite was split across a large mine site and a smaller mine site that was located out of the township but in the same region. The living arrangements of employees differed significantly between the two sites, with the majority of participants at the smaller site being fly-in fly-out workers (91.2%, *n* = 145), compared with the township site where the rate of fly-in fly-out was 21.3% (*n* = 186), χ^2^ (16) = 590.50, *p* < .001. No other significant differences were observed.

**Table 2:** Participant Characteristics

Characteristic	Frequency	Percent
Gender (*n* = 894)		
Male	658	73.6
Female	236	26.4
Age (*n* = 805)		
< 18 years	9	1.1
18–24 years	99	12.3
25–34 years	288	35.8
35–44 years	186	23.1
45–54 years	164	20.4
55–64 years	52	6.5
65–74 years	7	0.9
Marital status (*n* = 864)		
Partner	559	64.7
No partner	305	35.3
Employment status (*n* = 886)		
Employee	775	87.5
Contractor	111	12.5
Living arrangements (*n* = 888)		
Resident living in local to the mine site	702	79.1
Fly-in fly-out	186	20.9

*Note.* Values represent valid responses only

**Fig. 1:**
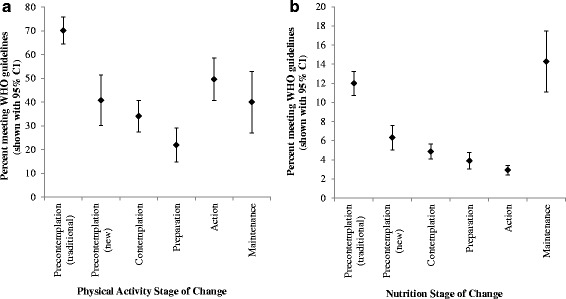
Percentage of employees meeting the World Health Organisation guideline for (**a**) physical activity and (**b**) nutrition by theoretical stage of change. Error bars represent 95% confidence intervals

A preliminary analysis of the association between theoretical stages of change and WHO health guidelines revealed overall significant differences in the percentage of participants who met the guidelines for physical activity (χ^2^ (5) = 101.89, *p* < .001, *Cramer’s V* = .35) and nutrition, χ^2^ (5) = 19.83, *p* < .001, *Cramer’s V* = .150. A further cross-tabulation of the percentage of sampled employees satisfying the WHO guideline was conducted (refer Table 3). Results of the cross-tabulation showed that the variance of participants who met the guideline was greater across the physical activity stages of change (range = 21.9% to 70.2%) when compared to nutrition (range = 2.9% to 14.3%). The percentage of participants who met the WHO guideline for either physical activity or nutrition, when compared to those who did not, was greater only in the group that identified with the traditional ‘precontemplation’ stage for physical activity. A visual inspection of the percentage of participants meeting the guidelines with 95% confidence intervals (shown in Fig. 1) revealed that the percentage of participants who met the physical activity guidelines was noticeably higher for those identified as being in the traditional ‘precontemplation’ stage of change for physical activity (*“As far as I’m concerned my exercise habits don’t need changing”*) than for individuals identified as being in any other stage of change for physical activity. Also evident in Fig. 1, the percentage of participants who met the nutrition guidelines was noticeably higher for those identified as being in the traditional ‘precontemplation’ stage for nutrition (*“As far as I’m concerned my eating habits don’t need changing”*) or the ‘maintenance’ stage for nutrition (*“I took action more than 6 months ago to change my eating habits and I’m working hard to maintain that change”*) than for individuals identified as being in any other stage of change for nutrition.

**Table 3:** Employees Meeting WHO Health Guidelines across Theoretical Stages of Change

Health Behaviour & Stages of Change	Meet Guidelines			
	No		Yes	
	*n*	%	*n*	%
Physical Activity (*n* = 828)				
Precontemplation (traditional)	74	29.8	174	70.2
Precontemplation (new)	48	59.3	33	40.7
Contemplation	130	66.0	67	34.0
Preparation	100	78.1	28	21.9
Action	60	50.4	59	49.6
Maintenance	33	60.0	22	40.0
Nutrition (*n* = 880)				
Precontemplation (traditional)	308	88.0	42	12.0
Precontemplation (new)	89	93.7	6	6.3
Contemplation	137	95.1	7	4.9
Preparation	74	96.1	3	3.9
Action	133	97.1	4	2.9
Maintenance	66	85.7	11	14.3

*Note.* Values represent valid responses only

## Discussion

This study compared a traditional and modified measure of the Stages of Change model with the health behaviours of physical activity and nutrition in a non-clinical workforce sample. As predicted, the modified survey measure of ‘precontemplation’ was more reliable at capturing participants who reported not engaging in the healthy behaviours of physical activity and nutrition, as assessed by the WHO guidelines [2]. This outcome supports the proposition that there is a strong need for validation of concise Stages of Change model measures for the assessment of healthy behaviour changes.

The Stages of Change model has traditionally been applied to unhealthy behaviours (e.g. smoking cessation) and the traditional survey measure reflects engaging in unhealthy behaviours, particularly at the lower end of the scale. For instance, according to the Stages of Change theory model, ‘precontemplators’ engage in the unhealthy behaviour and are unaware of that their current behaviour is problematic. Thus, the measure of ‘precontemplation’ should capture individuals that are not currently meeting the guidelines for healthy behaviours (such as physical activity and nutrition). The results demonstrated that the modified ‘precontemplation’ stage of change survey measures (“I know I should change my [exercise / eating] habits but I don’t intend to”) were associated with a reduced likelihood of capturing individuals who met the WHO guideline [2] for a healthy behaviour (as applied to physical activity and fruit and vegetable consumption) when compared to the traditional measure of *“As far as I’m concerned my [exercise / eating] habits don’t need changing”*. Therefore, it is considered likely that the modified ‘precontemplation’ measure was a more accurate assessment of individuals in the ‘precontemplation’ stage of change for a healthy behaviour.

Based on these findings, we know that participants who identified with the traditional ‘precontemplation’ stage for physical activity and / or nutrition *(“As far as I’m concerned my exercise / eating habits don’t need changing”*) were likely to meet the WHO guideline. For example, the results showed that as the stage of change increased, representing a theoretical increase in preparedness to change a health behaviour, the incidence of meeting the guideline for physical activity decreased, until the point of action. A similar trend was identified in the nutrition behaviour however the incidents of meeting the WHO guideline [2] did not increase until the measured maintenance stage. This suggests that either the survey measure lacks validity in the context of preparedness to change healthy behaviours or that the survey question was poorly understood by participants.

In Australia, where the study took place, the benefits of a healthy lifestyle are widely advertised through a variety of government campaigns and educational institutions. Accordingly, the modified wording indicated that the theoretical construct of ‘precontemplation’ for healthy behaviours within the Australian culture could better be represented as ‘individuals know that their behaviour is unhealthy but are apathetic to change’. Although the measure of ‘precontemplation’ was improved by the authors’ modification, it should be noted that both the traditional and modified survey question captured individuals currently satisfying the WHO guidelines [2] for healthy behaviours. This finding may reflect similar translation issues from unhealthy to healthy behaviours in the wording of the traditional survey measure of those within the ‘maintenance’ phase. Specifically, the traditional reference to changing a behaviour *“more than 6 months ago”* is not appropriate for persons who have never engaged in the unhealthy behaviour. It is likely that adjusting the ‘maintenance’ measure to include people that have sustained the healthy behaviour for an extended period of time may reduce the indices of healthy individuals identifying with lower stages of change for healthy behaviours.

Overall, participants in the ‘precontemplation’ stage of the model were more likely than those in the higher stages to meet the WHO guideline [2] for both physical activity and balanced dietary behaviours. This finding may represent a misunderstanding of the question by participants, but given the significant result it was considered more likely to indicate the need for refinement of the Stages of Change model survey measures for the assessment of healthy behaviours.

### Implications

This study provides practical contributions to both behaviour change theory and applied health promotion. Theoretically, employees who are classified as ‘precontemplative’, ‘contemplative’ or ‘in preparation’ should not be meeting the health guidelines. However, this study identified that the current stages of change measure incorrectly classified some employees who met the guidelines as ‘precontemplative’, ‘contemplative’ or ‘in preparation’. Similarly, some employees who did not meet the guidelines were incorrectly classified as being in the ‘action’ or ‘maintenance’ stage. This finding implies that further refinement is required to achieve concise measures of theoretical stages of change that can be applied to healthy behaviours. The development and validation of concise measures that reliably classify employee readiness for engaging in behaviours that meet physical activity and nutrition guidelines is essential to facilitate the appropriate matching of semi-tailored obesity risk management strategies with an individual’s readiness. Furthermore, previous behaviour change findings in which the theory has been applied to risky behaviours may not be generalised to healthy behaviours.

In addition, this paper makes a valuable contribution to public health research and practice. Specifically, the rising prevalence of obesity rates suggest that current mass education campaigns are, by themselves, ineffective as a public health prevention strategy. Understanding the differences in cognitive appraisals of healthy lifestyle behaviours and how to assess them, as highlighted in this study, may be used to inform the development of semi-tailored obesity prevention strategies targeting individuals who are most at need of such interventions, namely ‘precontemplators’. Thus, it is anticipated that improving the scientific validity of measuring behaviour change intentions and development of workplace and public health campaigns as outlined herein will result in more effective preventative health promotion strategies that target healthy lifestyle behaviours and obesity.

### Limitations

Limitations of the current study included the reliance on self-report measures for existing health behaviours, potentially insufficient literacy skills of some participants, cluster volunteer sampling methodology, and possible gender influence on participants’ healthy lifestyle behaviours.

Ideally, complex health behaviours such as physical activity and nutrition require more objective measures (e.g. through the use of an activity tracker device). Regrettably, organisational constraints and the large workforce sample meant that objective measures were not considered feasible by the organisation in this instance.

Whilst conducting the health survey the researchers were informed by Managers that a small number of participants did not have the literacy skills to complete the survey. When informed of these instances, the researchers assisted by reading the questions aloud to the participant and transcribed their responses in order to overcome this barrier. However, it is possible that some additional individuals with poor literacy skills did not inform the researchers and may have completed the survey without fully understanding the questions. Furthermore, for those who had the questions read aloud, this action may have resulted in a bias or Hawthorne effect whereby the participant responded with the answer they assumed the researcher expected to hear.

In order to minimise disruption to the operational processes of the organisation, cluster volunteer sampling was employed whereby employees of selected work units were invited to participate. Although consideration was given to selecting a variety of work units that represented a broad spectrum of employees, it is not possible to ascertain whether differences in demographic characteristics, health behaviours, or stage of change would have been observed for employees in work units that were not included.

Finally, it should be acknowledged that the sample included a high proportion of males (73.6%) and that the potential influence of gender on health behaviours and preparedness to change was not investigated in this study. Therefore, it is unclear whether the results presented herein are generalisable to a population sample where the gender proportions are more balanced.

### Future research

Despite the aforementioned design limitations of the current study, including the potential for gender effects on lifestyle behaviours and preparedness to change, the findings highlight the ineffectiveness of current measures and should be used as a baseline study to inform future research into behaviour change measurement for healthy behaviours and public health campaigns targeting obesity. More specifically, future research should work to refine the measurement of the Stages of Change model for application to healthy behaviours in a general population. It is recommended that researchers refine the concise measures of stages of change to include definitions of physical activity and nutrition guidelines in the question and response options that specify yes and no with regards to meeting the guidelines in addition to readiness for change options. For example, the concise nutrition stages of change measure could ask *“On average do you eat at least five serves of vegetables and two serves of fruit daily?”* An example response option for the modified ‘precontemplation’ stage that incorporates reference to not meeting the guideline could be *“No, I know I should improve my eating habits but I don’t intend to”*. Future health behaviour change studies should not only consider, but also evaluate the role of behaviour change theory in the program development process.

## Conclusions

This study further extended the Stages of Change model by including an additional measure of ‘precontemplation’. A self-report health survey incorporating a modified stages of change measure of ‘precontemplation’ was developed as part of this project. The survey was administered onsite to 897 shift workers in rural Australia. Results of the analyses revealed that the modified measure of ‘precontemplation’ better reflected the theoretical construct. However, further refinement and evaluation of the Stages of Change model is required for application to healthy behaviours, compared to health risk behaviours. The results also revealed the lower stages of change were associated with higher levels of adherence to the WHO guidelines [2], but this trend reversed at the action stages for physical activity and maintenance stage for nutrition behaviours. Accordingly, the results of this study have important theoretical implications for advancing the measurement of psychological preparedness for healthy behaviour changes. The findings also have practical contributions and can be applied to inform the design and implementation of behaviour change strategies to proactively reduce obesity related preventable disease and premature deaths.

## Abbreviations

URICA: University of Rhode Island Change Assessment Scale
WHO: World Health Organization
